# Coronal and heliospheric magnetic flux circulation and its relation to open solar flux evolution

**DOI:** 10.1002/2016JA023644

**Published:** 2017-06-05

**Authors:** Mike Lockwood, Mathew J. Owens, Suzanne M. Imber, Matthew K. James, Emma J. Bunce, Timothy K. Yeoman

**Affiliations:** ^1^ Department of Meteorology University of Reading Reading UK; ^2^ RAL Space Rutherford Appleton Laboratory Chilton UK; ^3^ Space and Atmospheric Physics Group, Blackett Laboratory Imperial College London London UK; ^4^ Department of Physics and Astronomy University of Leicester Leicester UK

**Keywords:** solar magnetic field, open solar flux, solar magnetic cycle, meridional transport

## Abstract

Solar cycle 24 is notable for three features that can be found in previous cycles but which have been unusually prominent: (1) sunspot activity was considerably greater in the northern/southern hemisphere during the rising/declining phase; (2) accumulation of open solar flux (OSF) during the rising phase was modest, but rapid in the early declining phase; (3) the heliospheric current sheet (HCS) tilt showed large fluctuations. We show that these features had a major influence on the progression of the cycle. All flux emergence causes a rise then a fall in OSF, but only OSF with foot points in opposing hemispheres progresses the solar cycle via the evolution of the polar fields. Emergence in one hemisphere, or symmetric emergence without some form of foot point exchange across the heliographic equator, causes poleward migrating fields of both polarities in one or both (respectively) hemispheres which temporarily enhance OSF but do not advance the polar field cycle. The heliospheric field observed near Mercury and Earth reflects the asymmetries in emergence. Using magnetograms, we find evidence that the poleward magnetic flux transport (of both polarities) is modulated by the HCS tilt, revealing an effect on OSF loss rate. The declining phase rise in OSF was caused by strong emergence in the southern hemisphere with an anomalously low HCS tilt. This implies the recent fall in the southern polar field will be sustained and that the peak OSF has limited implications for the polar field at the next sunspot minimum and hence for the amplitude of cycle 25.

## Key Points

- The tilt of the heliospheric current sheet is shown to modulate the loss rate of open solar flux
- Not all magnetic flux emerged in active regions contributes to the solar cycle evolution of solar polar regions
- The rise in open flux in the declining phase of cycle 24 was due to reduction of the loss rate as well as increase in the production rate

## 1 Introduction

The importance of flux transport in the solar photosphere and corona was first recognized by *Leighton* [1964], as discussed in a review of the development of the concepts by *Sheeley* [2005]. Evolution of photospheric flux is predicted by numerical models which follow magnetic flux tubes that have emerged through the photosphere in “bipolar magnetic regions” (BMRs) [*Harvey and Zwaan*, 1993] and evolve under the combined effects of differential rotation, meridional flow, and diffusion resulting from quasi‐stochastic granular and supergranular motions. These motions in the photosphere are plasma dominated (the plasma *β* parameter being high) and essentially two dimensional. The development of photospheric flux transport models was through a long series of contributions [e.g., *DeVore et al.,* 1984; *Sheeley et al.,* 1985, 1987; *Wang and Sheeley,* 1989; *van Ballegooijen et al.,* 1998]. The photospheric flux transport sets the inner boundary conditions for the evolution of the coronal magnetoplasma which is dominated by the magnetic field (low *β*) and is three dimensional in nature. The coronal field evolves through advection, the expansion/contraction of field line loops, and magnetic reconnections which exchange photospheric field line foot points and facilitate removal of magnetic flux and helicity through the outer coronal boundary (i.e., into the solar wind) [*Wang et al.,* 2000; *Mackay et al.,* 2002; *Mackay and Lockwood,* 2002; *Schrijver et al.,* 2002; *Mackay and Yeates*, 2012; *Jiang et al.,* 2014].

Whereas photospheric fields can be measured by magnetographs from Zeeman splitting of emission lines, coronal fields have to be inferred from images of plasma on field line loop structures and/or modeled from the knowledge of the field in the photosphere. The most commonly used model is the “potential field source surface” (PFSS) [*Schatten et al.,* 1969; *Altschuler and Newkirk*, 1969; *Schatten*, 1999], which makes a number of assumptions. This involves solving Laplace's equation within an annular volume above the photosphere in terms of a spherical harmonic expansion, the coefficients of which are derived from Carrington maps of the photospheric magnetic field (i.e., maps assembled over an entire solar rotation): hence, a major assumption is that there are no temporal variations within the 27 days taken to build up the map. This method also assumes that there are volume currents but no sheet currents in the corona (an assumption needed so as to allow unique solutions in closed form). To eliminate the possibility that such simple harmonic expansions would result in all of the magnetic field lines returning to the Sun within a small heliocentric distance, the outer coronal field is required to be radial. Despite its many assumptions and obvious limitations, PFSS has been very helpful in the study of a wide range of solar and heliospheric phenomena [see review by *Mackay and Yeates*, 2012]. In essence, PFSS yields the equilibrium configuration that the corona is heading toward for a given distribution of photospheric field rather than the time‐dependent corona that is evolving toward that equilibrium: “magnetofrictional” models [*Yeates et al.,* 2008, 2010] have been developed to try to address this and often use photospheric flux transport models to give input Carrington maps of photospheric field of greater time resolution than 27 days. Note that in the present paper we do make use of PFSS but only in one context and that is to quantify the tilt of the main heliospheric current sheet (HCS): this application will be discussed in section 2.

Some of the basic mechanisms behind the 22 year “Hale” cycle evolution of solar magnetic fields were proposed by *Babcock* [1961], *Leighton* [1969] and *Parker* [1955]. Each sunspot cycle begins with a poloidal seed field which threads the solar interior and is seen in the photosphere and corona near the poles of the Sun. Differential rotation converts this poloidal field into a toroidal configuration somewhere below the photosphere. The toroidal field generated has opposite senses in the two hemispheres. Thus, when the field rises up through the photosphere (emerges) in BMR loops, the leading associated sunspots (and active‐region faculae) will have opposite field polarities in the two hemispheres, as is observed (Hale's polarity law). These polarities reverse with each new solar cycle, telling us that the toroidal field has swapped polarity, as has the initial “seed” poloidal field. Because of buoyancy considerations for the large field strengths seen in BMRs, generation and storage of the toroidal field is now thought to take place in an “overshoot layer” just below the convection zone proper where the differential rotation of the convection zone penetrates down into the top of the radiative zone [e.g., *Nandy and Choudhuri*, 2002].

As the flux rises through the convection zone, plasma parcels and their frozen‐in toroidal field are twisted by the Coriolis force (the “*α* effect”, introduced by *Parker* [1955]), which generates a north‐south (poloidal) component from the erupting toroidal field and causes the trailing sunspots to be at a higher latitude than the leading spots, as is observed for most BMRs (Joy's law). The angle between the line connecting the centers of linked spots of opposite polarity and the line of constant heliographic latitude, caused by the *α* effect, is called the “sunspot tilt angle.”

These early studies of the behavior of solar magnetic fields do not, by any means, add up to a full understanding of their evolution and behavior. However, they do give insights that are useful to the present study. First, emergence of BMRs peaks at middle latitudes where the latitudinal gradient of rotation rate is greatest (and is low at the solar rotational (heliographic) equator where this gradient is zero). This means that most BMRs are formed with foot points in the same solar hemisphere and other processes are required to generate any open flux loops that have one foot point in each hemisphere. Second, the sunspot tilt angle means that the field of the polarity of trailing sunspots is generally at higher latitudes and so it is harder for that polarity to migrate against the poleward meridional flow (see next paragraph) and move into the opposite hemisphere. This paper will investigate the significance of these insights for OSF evolution.

As the solar cycle progresses, the magnetic field of active regions is dispersed by turbulent convection and tends to migrate poleward under the meridional flow of the foot points rooted in the upper convection zone [*Giles et al.,* 1997; *Wang et al.,* 2002] and also via foot point exchange of field lines caused by reconnection with small loops of flux [*Wang and Sheeley*, 2004]. These transport mechanisms lead to the accumulation of magnetic flux of the polarity of the trailing spots at high solar latitudes, eventually reversing the polarity of the polar fields which therefore then have the right sense to be the “seed field” for the next solar cycle. While this evolution from BMRs to poloidal polar flux is ongoing, the loops also rise through the solar corona, emerge through an arbitrarily defined surface at the top of the corona, and enter the heliosphere, where they are “frozen‐in” in the supersonic and super‐Alfvénic solar wind and so dragged away from the Sun. The surface is called the “coronal source surface” and is often taken to be a heliocentric sphere of radius *r* = 2.5 *R*_**⊙**_, where *R*_**⊙**_ is a mean solar radius: in the PFSS modeling, this is where the field is assumed to be radial. Flux that has emerged through the coronal source surface is defined to be “open solar flux” (OSF). Observationally, new OSF appears to be primarily the result of closed loops dragged out by eruptive coronal mass ejection (CME) activity [*Owens and Crooker,* 2006, 2007]: although rising ambient coronal loops would serve the same purpose and their contribution is difficult to quantify. The total OSF rises and falls over each solar cycle and also shows long‐term secular variation [*Lockwood et al.,* 1999, 2009b].

It has been demonstrated empirically that the magnitude of the polar field at each sunspot minimum controls, somewhat directly, the magnitude of the subsequent solar cycle [*Svalgaard et al.,* 2005; *Petrovay*, 2010; *Schatten and Pesnell*, 2012; *Muñoz‐Jaramillo et al.,* 2013; *Cameron et al.,* 2014], and this is the basis of the several empirical methods for predicting the amplitude of the upcoming sunspot cycle based on solar‐terrestrial conditions during the prior sunspot minimum. This being the case, the variability of solar activity from one cycle to the next must arise from variability in the percentage of the flux emerging through the photosphere in sunspot regions that is able to evolve all the way to the polar coronal holes at the end of the cycle. Thus, there has been much recent debate as to the origin of this variability. *Wang et al.* [2002] discussed the role of variations in the speed of the meridional circulation. The numerical flux transport models [e.g., *Mackay et al.,* 2002, *Mackay and Lockwood,* 2002] also show effects of sunspot tilt angle because it influences the foot point velocity difference caused by differential rotation, and *Cameron et al.* [2014] investigated the role of sunspot tilt angle on polar field growth and hence on the subsequent solar cycle. *Dasi‐Espuig et al.* [2010] had found a relation between the mean active region sunspot tilt angle of a given cycle and the strength of next cycle, a result that was questioned by *McClintock and Norton* [2013]. *Dasi‐Espuig et al.* [2013] did correct their results which weakened the anticorrelation found somewhat but did not remove it.

In this paper we present evidence that other factors also contributed to the variability in the polar OSF.

## 2 Flux Circulation in the Corona and Heliosphere

**Figure 1:**
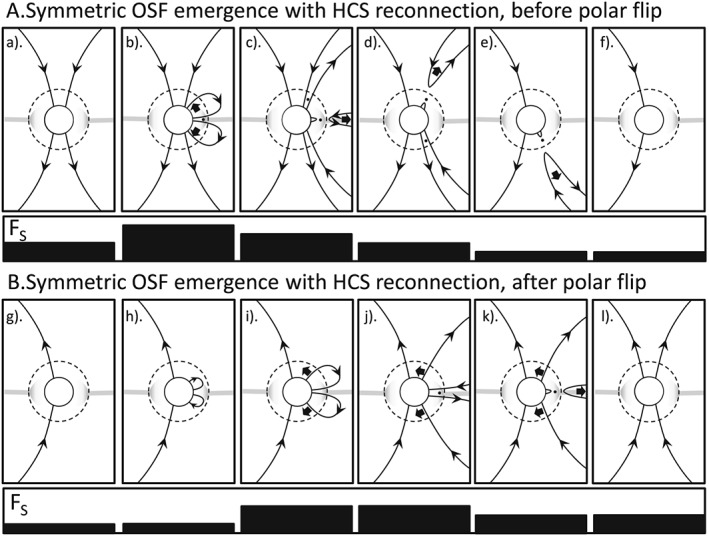
Schematics of the evolution of magnetic flux in heliographic latitude and heliocentric distance. North is to the top of each panel. The solid circle represents the solar photosphere and the dashed circle the critical surface where the flow becomes super‐Alfvénic. The grey line is the heliospheric current sheet (HCS) which breaks up into a network of smaller sheets in the solar corona. Note that the HCS is shown at low latitudes not because solar activity is considered to be low (when the HCS flat and quite well aligned with the solar equator) but because Carrington longitudes have been selected for display at which the HCS crosses the solar equator: this is because both theory and observations show that the OSF disconnection rate in the HCS will peak there because of the large inclinations of the HCS on the Carrington latitude‐longitude map at such points. Arrows on lines give the field direction; solid arrows give the motion of the field lines and the dots reconnection sites at which the solar wind flow is sub‐Alfvénic (so that disconnected flux is carried out into the heliosphere and the connected loop collapses back toward the Sun). Sequence A (panels a to f) is for early in solar cycle 24 before the polar field has flipped direction; Sequence B (panels g to l) is after this time. For both sequences A and B, the long panel underneath the series of schematics shows the variation of the open solar flux that threads the coronal source surface, F_S_, in each schematic. This case is for emergence of flux through the photosphere and source surface that is symmetric in the northern and southern hemispheres and with a matching reconnection rate in the HCS.

**Figure 2:**
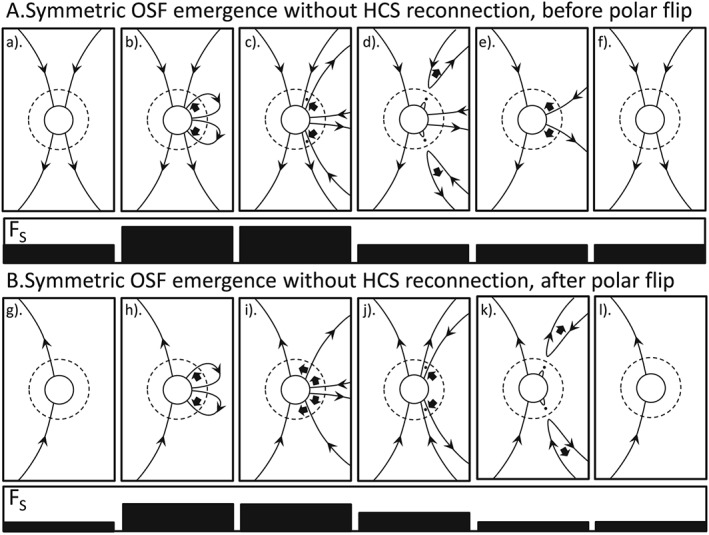
Same as Figure 1, for the case of emergence of flux through the photosphere and source surface that is symmetric in the northern and southern hemispheres but in the absence reconnection in the heliospheric current sheet (HCS). No reconnection at the HCS is involved, and so it is not shown.

**Figure 3:**
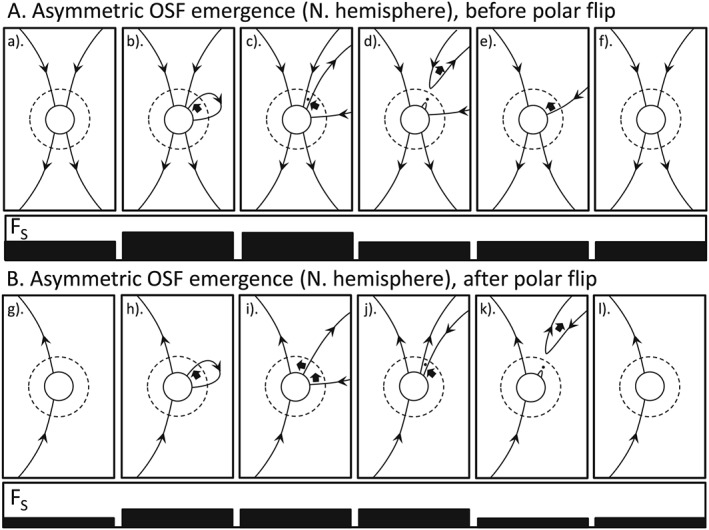
Same as Figure 1, for the case of asymmetric emergence with all flux emergence through the photosphere and source surface in the northern hemisphere. In this case there can be no reconnection in the heliospheric current sheet (HCS).

**Figure 4:**
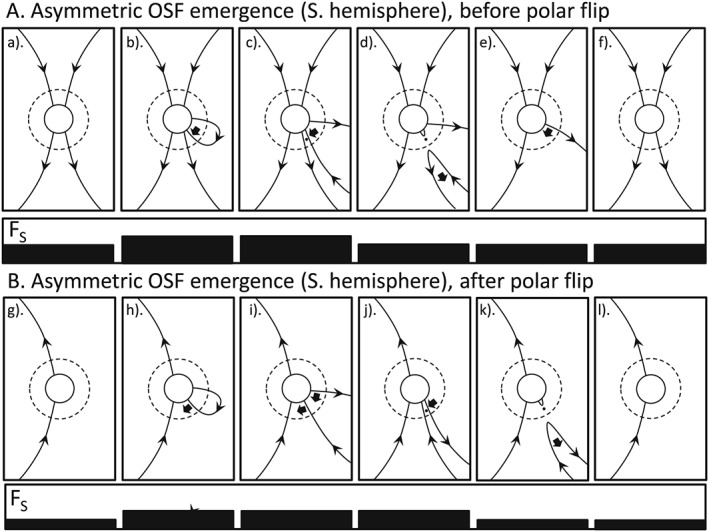
Same as Figure 3, with all flux emerging through the photosphere and source surface in the southern hemisphere.

**Figure 5:**
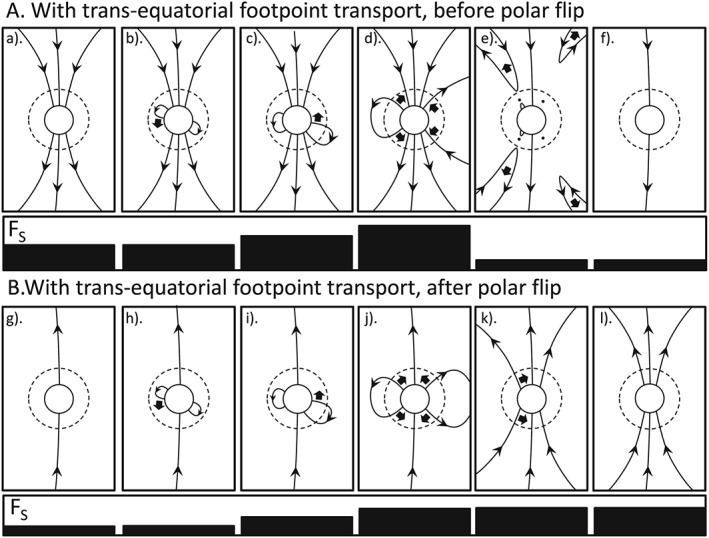
Similar to Figure 1 but showing the evolution of loops that emerge in one hemisphere but evolve into having foot points in opposing hemispheres through transequatorial foot point exchange with closed coronal loops or with several interchange reconnections with the very small loops of the magnetic carpet. Two variations of this are shown occurring roughly simultaneously on the two limbs of the Sun: on the right‐hand limb the loop evolves into open flux before the foot point exchange(s) give photospheric foot points in both hemispheres and on the left‐hand limb the loop only becomes open after it has evolved to have foot points in opposing hemispheres. Both sequences advance the solar cycle evolution of the polar field.

The possible latitudinal evolution paths of open solar flux (OSF) were illustrated, collectively in the schematic Figure 36 of *Lockwood* [2004]. Figures 1, 2, 3, 4, 5 of the present paper consider the possible classes of evolution path and the variations in OSF that each contributes, individually. It must be noted that these are clearly two‐dimensional sketches of an inherently three‐dimensional solar corona and that longitudinal structure will almost certainly add complexity. However, in this paper, it is never assumed that the field line loops lie in meridian planes and so the fact that, in reality, they have various extents in the longitudinal direction is of no consequence and does not influence the arguments made.

Figure 1 shows the effect of OSF emergence that is the same in both the northern and southern hemispheres and at a rate which is matched by the rate of flux disconnection by magnetic reconnection taking place in the heliospheric current sheet (HCS). The upper sequence (part A) shows the evolution for the rising phase of cycle 24, and the lower sequence (part B) is that for the declining phase of the cycle, after the polar fields have flipped polarity. For sequence A, the polar fields point toward/away from the Sun in the Northern/Southern Hemispheres but the converse is true for sequence B. In each frame, the solid circle is the photosphere and the dashed circle is at *r* = *r*_C_, the critical heliocentric distance where the solar wind flow becomes super‐Alfvénic. The grey line in Figure 1 is the HCS. Note that the schematics in Figure 1 select Carrington longitudes at which the HCS is near the solar equator and that at other longitudes the HCS will be at higher latitudes (especially around sunspot maximum). The reason for this choice is discussed below. The HCS is shown as extending as a coherent single sheet outside the source surface (which is generally taken to be at *r* = 2.5 *R*_**⊙**_ whereas *r*_C_ is typically between 10 *R*_**⊙**_ and 20 *R*_**⊙**_). Below the source surface, the HCS increasingly breaks up into a network of smaller current sheets with decreasing *r* and so is depicted as a broadening and in lighter grey (as current densities decrease). This is as envisaged by *Antiochos et al.* [2011] when discussing the origins of the slow solar wind. The OSF contribution of the field lines shown in each schematic is given in the panels underneath both sequence A and sequence B. A fraction of the field that emerges through the photosphere in BMRs evolves upward to thread the coronal source surface becoming, by definition, OSF. This is shown happening in Figures 1b, 1h, and 1i. In the case presented in Figure 1, all these emerged OSF loops reconnect where opposite polarity field meets at the HCS (grey line). The only relevant reconnection takes place at heliocentric distances at which the solar wind flow is sub‐Alfvénic (i.e., at *r* < *r*_C_) which results in the disconnected flux being dragged out into the heliosphere by the solar wind and the sunward loop collapsing back toward and below the photosphere, against the solar wind flow and under the action of the magnetic curvature force. These sunward flow events have been observed in coronal images and have been interpreted in terms of flux disconnection events [*Sheeley and Wang*, 2002, 2007]. These disconnections halve the newly emerged OSF. The importance of the magnetic reconnection taking place close enough to the Sun for the flow to be sub‐Alfvénic is that, otherwise, the loop to the sunward side of the reconnection site is also pulled away from the Sun and the OSF is not altered: indeed, because in such cases no information about the reconnection could propagate back to the source surface or photosphere, it would have no relevance to the evolution of coronal or photospheric fields.

Note that in Figure 1 the HCS disconnection events are shown taking place at Carrington longitudes where the HCS is close to the solar equator even though, at sunspot maximum at least, the HCS covers a wide range of heliographic latitudes. This is because it is the angle of the HCS on a Carrington latitude‐longitude map (i.e., relative to the direction of solar rotation motion) that matters for the disconnection of OSF because it brings open flux of opposite polarity into longitudinal proximity and that HCS tilt peaks at low latitudes (and is zero at the peaks of the HCS excursion from the solar equator) [*Owens et al.,* 2011a]. At all latitudes there must be foot point‐changing reconnections taking place between open field lines and their longitudinal neighbors to allow the corona to rotate differently to the photosphere [*Nash et al.,* 1988; *Wang and Sheeley*, 1993]. If the open field line is surrounded by other open field lines of the same polarity (i.e., away from the HCS), the reconnection will be with longitudinal neighboring opposite polarity flux of closed loops that do not emerge through the coronal source surface and may be the very small loops of the “magnetic carpet” close to the photosphere [*Schrijver and Title*, 2003], giving photospheric foot point motion, and the effect is just to allow the corona to rotate at a different rate to the photosphere below. If, however, the longitudinal neighbor is open and has the opposite polarity (i.e., the HCS runs between them), then disconnection will occur. For this reason, we here use the “current sheet tilt index,” *I*_HCS_, (the fraction of pixels in which the (open) field at the coronal source surface has the opposite polarity to its longitudinal neighbor), as a proxy to quantify the rate of HCS disconnection of OSF [*Owens et al.*, 2011a]. To derive *I*_HCS_, we make use of PFSS, which as discussed in section 1 makes several approximations and assumptions: to understand their implications, we note that the main HCS, as derived by PFSS, matches well with that derived from averaged magnetometer data in the heliosphere from spacecraft such as Ulysses [see, e.g., *Owens and Forsyth,* 2013, Figure 13]. Note, however, that PFSS could not detect the smaller‐scale, time‐dependent current sheets that are seen in higher time resolution Ulysses data (see below) and predicted by nonpotential models of the corona [*Yeates et al.,* 2010] and which are invoked in Figures 1, 2, 3, 4, 5. PFSS is only used here to quantify the tilt of the main HCS and is not invoked in the conceptual models that we discuss. The low latitudes of these OSF disconnection events is supported by the observations of coronal inflows in coronagraph data, thought to be their low‐altitude signature, which occur where the HCS tilt is greatest (i.e., preferentially at low latitudes) [*Sheeley and Wang*, 2002, 2007]. Hence, in Figure 1 the longitudes shown are those at which the HCS crosses the solar equator as it at such longitudes where OSF disconnection occurs most often. The modulation of reconnection by HCS tilt is here taken only to apply to OSF. Although there is no known reason to expect this effect to cease completely at the source surface (meaning it will, to some degree, extend to the current sheets into which the HCS divides below the coronal source surface), there is also no evidence that it does and we do not assume that it reaches all the way down to the photosphere and consider its effect to only be on OSF.

The photospheric foot points of the remaining newly emerged OSF then migrate poleward under the joint effect of meridional circulation [*Giles et al.,* 1997; *Wang et al.,* 2002] and foot point exchange [*Wang and Sheeley*, 2004]. In sequence A (Figures 1a–1f), this brings them into contact with the polar field left over from the previous solar cycle which is in the opposite direction. Near‐Sun reconnection between the two then allows high‐latitude disconnection. In Figures 1d and 1e this is shown as taking place in the northern and then the southern hemisphere: the order is not important as the net effect is to reduce the pre‐existing polar flux.

In Part B (Figures 1g–1l), the sequence of events show in Part A has removed all the pre‐existing (old polarity) polar field and started to add flux of the new polarity. The same sequence of emergence, HCS reconnection, and poleward transport occurs in in Part B as in Part A, but the sequence shown in B is adding to the new polarity flux collecting at the poles. In general, both polarities could coexist for a while and the polar field flip is when the average field polarity changes.

Note that in Figure 1, one of the high‐latitude reconnections could take place before the HCS reconnection, especially for very large emerging loop structures in CMEs. In such examples the first reconnection would cause the OSF foot point to jump down to low latitudes (foot point exchange) where it would then be disconnected by the HCS reconnection. The time series of the OSF variation would be exactly the same, but it would cause low‐latitude coronal holes to exist for the interim period between the two reconnection events. Such a sequence of is a likely to be involved in the production of the “coronal fans” seen in images of the corona by Sun Watcher with Active Pixels and Image Processing EUV telescope on board the PROBA2 satellite [*Seaton et al.,* 2013].

A key point about both sequences shown in Figure 1 is that they move the solar cycle forward, in terms of the evolution of the polar fields: in part A the sequence is contributing to the decay of the remnant flux from the previous solar cycle and in part B it is building up the new polarity polar coronal holes that will be seen in full at the next sunspot minimum.

Notice that Figures 1, 2, 3, 4, 5 all invoke current sheets in the corona and heliosphere that are well removed from the main HCS. The existence of such current sheets is consistent with data from the heliosphere. For example, *Balogh et al.* [1999] report a polar current sheet seen by the Ulysses spacecraft that is well removed from the main HCS. Indeed, during its second perihelion pass (“fast‐latitude scan” that took place between December 2000 and October 2001 and so near sunspot maximum), the Ulysses spacecraft detected 1024 radial field polarity reversals (i.e., current sheets, both tangential, and rotational discontinuities) in hourly means of the radial field between heliographic latitudes 80° and +80° [*Lockwood et al.,* 2004]. The same data averaged into daily means yield 52 such polarity reversals (and some of them are bunched together, and these look like structure in the main HCS or motions of the HCS back and forth over the satellite). When averaged over 3 days, the data show just 23 polarity reversals in the perihelion pass, two for every full solar rotation. Thus, the classic tilted structure of the main HCS emerges only when averaging over 3 days. Obviously, not all of the 1024 current sheets seen in the hourly data map back to solar corona (many will be generated in the heliosphere by Alfvén waves, or stream‐stream interactions or CME transients), but some of them could map back to the corona or be at the center of disconnected U‐shaped field lines that are fossil remnants of reconnection closer to the Sun in current sheets that may no longer be present at the location. Hence, although not part of the main HCS, the additional current sheets in Figures 1, 2, 3, 4, 5 are not inconsistent with the Ulysses data.

Figure 2 shows the same symmetric emergence as in Figure 1, but for flux that does not undergo transhemisphere reconnection in the HCS. Note that this can be taking place at the same time as the evolution shown in Figure 1 and applies to any flux generated too quickly to be reconnected at the HCS, and so evolves away from it instead, or to flux at longitudes where the HCS is not inclined to the solar rotation direction (e.g., at the peaks of HCS variation away from the solar equator). Such flux will migrate poleward, but possibly at a rate slower than for the flux of the trailing (higher latitude) field polarity of the BMR. This is because the poleward migration for this leading spot polarity would be due to the meridional circulation: foot point exchange would be dominated by reconnection with BMRs and small remnant loops that, on average, obey Joy's polarity law which would mean that they would tend to oppose the poleward migration because they are biased toward causing the leading polarity foot points to jump to a lower, rather than higher, latitudes. Also, observations indicate that the poleward meridional flow is slower closer to the equator. The speed with which magnetic features migrate poleward in the photosphere and the speed of poleward meridional circulation flows (as observed by Doppler measurements and, where possible, helioseismology) are found to be similar [*Hathaway and Rightmire*, 2011; *van Ballegooijen et al.,* 1998; *Schrijver and Title*, 2001; *Ulrich*, 2010; *Zhao and Kosovichev*, 2004; *Švanda et al.,* 2007], but foot point exchange means that OSF will migrate poleward at a different speed to the photospheric field and will show sudden jumps if the closed coronal loops involved are large. Any OSF with the polarity of the leading spots that is not moved back to the HCS and there undergo near‐Sun reconnection, will evolve poleward under meridional circulation.

In Figure 2, there is poleward migration of both polarity foot points in both hemispheres and eventually these meet at or near the poles and disconnection takes place via high‐latitude reconnection. In Figures 2a and 2b, this returns the polar OSF to where it was before the sequences started. In other words, for both before and after the polar field flip, this sequence (the same as in Figure 1 but without near‐Sun reconnection in the HCS) causes a rise and the fall in the OSF but does not progress the cycle in terms of the solar cycle evolution of the polar fields. Note also (not shown) that the emerged loop foot points could reconnect with each other, meaning that its flux was added to the OSF for an even more brief interval, being disconnected before either foot point reaches the polar cap.

Figures 3 and 4 deal with OSF emergence that is asymmetric such that OSF emerges in one hemisphere that is not matched by emergence in the other hemisphere. In Figure 3 the excess emergence is in the northern hemisphere; in Figure 4 it is in the southern. Because the excess is not matched by any flux on the other side of the HCS, it cannot reconnect there. The evolution is then the same as in that hemisphere in Figure 2. Again, these two sequences cause a rise and then equal fall in the OSF but do not contribute to the solar cycle evolution of the solar polar flux.

Hence, of the sequences discussed in Figures 1, 2, 3, 4, only that in Figure 1 advances the solar cycle in terms of the evolution of the polar fields. However, as discussed by *Petrovay* [2010], *Cameron et al.* [2013], and *Owens et al.* [2007], there is another class of sequence that achieves this and this is shown in Figure 5. It is similar to Figure 1 in that the key element is that foot point‐exchanging reconnections generate open flux that has foot points in opposite solar hemispheres, i.e., loops that straddle the heliographic equator and so have foot points that are embedded in meridional flows toward opposite poles. The difference is that the reconnection(s) happen earlier in the sequence and need not be in the HCS. Instead, a photospheric foot point is passed across the heliographic equator (and potentially under the inner edge of the HCS) by foot point exchanging reconnections in the lower corona. This is illustrated in Figure 5. Two variants of this class of sequence are shown occurring roughly simultaneously on the two limbs of the Sun: on the right‐hand limb the loop evolves into open flux before the foot point exchange(s) give photospheric foot points in both hemispheres (this was discussed in relations to coronal mass ejection OSF loops by *Owens et al.* [2007]); on the left‐hand limb the loop only becomes open after it has evolved to have foot points in opposing hemispheres. The foot point exchange across the solar equator could be achieved with single reconnection with a BMR formed straddling the equator, but these are very rare (and only seen late in the solar cycle) and so is more likely to be achieved via a series of reconnections with the very small loops of the magnetic carpet. Both sequences advance the solar cycle evolution of the polar field. Note that there is no known reason to consider the sequences that advance the solar cycle (shown in Figures 1 and 5) mutually exclusive: both could be ongoing simultaneously at different Carrington longitudes and/or they could both occur at different times. In this paper we are certainly not suggesting that sequences like those shown in Figure 5 do not occur: we concentrate on those shown in Figure 1 because they have the ability to explain some unusual features of cycle 24.

However, one point to note about the sequences in Figure 5 is that if they were the only ones to occur, then all OSF that emerged would migrate to the polar caps which, given the strong correlation between the polar fields and the amplitude the subsequent sunspot cycle, would mean that most of cycle‐to‐cycle variability in solar magnetic activity would arise from variations in the fraction of emerged photospheric flux that also emerges through the coronal source surface and becomes open. However, reconstructed long‐term variations show a strong relationship between the cycle amplitudes in OSF and sunspot number [*Lockwood et al.,* 1999, 2014; *Lockwood*, 2013] and hence that the variability in the fraction of emerged flux that becomes OSF is low. Thus, the sequences shown in Figure 5 would not, on their own, give an explanation of cycle‐to‐cycle variability in solar magnetic activity, but variations in the degree to which they occur relative to the sequences in Figures 2, 3, 4 (that do not advance the solar cycle) would. The same is true for the other sequence that does advance the solar cycle, namely, that illustrated by Figure 1.

## 3 Solar Cycle 24 and Observations of the Heliospheric Field Near Mercury and Earth

**Figure 6:**
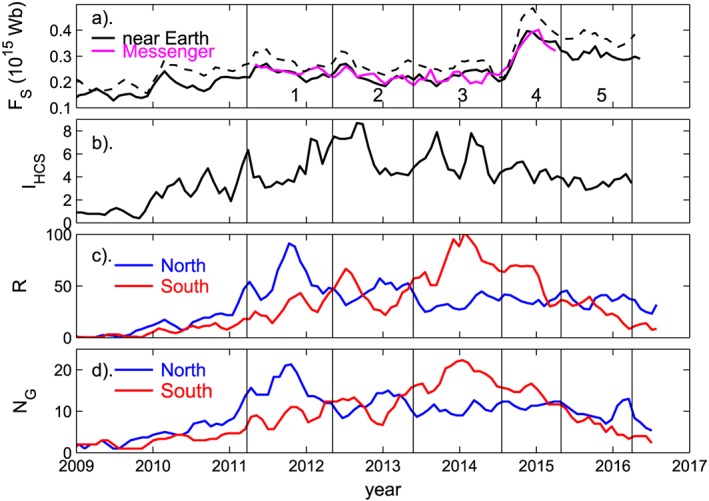
Time series of various parameters during solar cycle 24. In all panels, 3‐point running means of Bartels rotation period averages are shown. (a) The signed open solar flux, *F*_S_, from near‐Earth data (in black) and MESSENGER data (in mauve). In both solid lines the source flux is computed using a *r* ^−2^ dependence and the kinematic correction to allow for the effect of velocity shears on the radial field. The dashed line gives the near‐Earth values if the kinematic correction is not made for the means of the modulus of hourly means of the radial field and an *r* ^−2^ dependence assumed. (b) The heliospheric current sheet tilt index, *I*_HCS_. (c) The hemispheric sunspot numbers, *R*_N_ (for the northern hemisphere, in blue) and *R*_S_ (for the southern hemisphere, in red). (d) The hemispheric sunspot group numbers, *N*_GN_ (for the northern hemisphere, in blue) and *N*_GS_ (for the southern hemisphere, in red). The vertical black lines delineate five intervals that are compared in this paper (see text for details).

The time series shown in Figure 6 display the features of cycle 24 which, while present in many other solar cycles, have been particularly prominent during the current cycle. Figure 6a shows the open solar flux (OSF) from spacecraft data: the feature of note is that after a modest rise in the first half of the cycle, there is a strong rise early in the declining phase after the polar field flip [*Wang and Sheeley,* 2016a, 2016b]. Figure 6b shows the heliospheric current sheet (HCS) tilt index, *I*_HCS_, introduced by *Owens et al.* [2011a]. This is defined from PFSS mapping of observed photospheric field to the coronal source surface. For convenience, *I*_HCS_ has here been defined as 10^3^ *f*_SS_, where *f*_SS_ is the fraction of source surface grid cells which have opposite field polarity to their immediate longitudinal neighbors. Hence, *I*_HCS_ quantifies the fraction of the grid cells where the HCS (when quantised into grid cells) is locally perpendicular to the rotation direction and is designed to be a proxy indicator of the OSF disconnection rate at the HCS. Over cycle 24, *I*_HCS_ varies in a similar way to that derived for previous solar cycles [*Owens and Lockwood*, 2012] in that there is a relatively sharp rise followed by a more gradual fall. The unusual feature is that there are some relatively prominent fluctuations in *I*_HCS_ (as discussed below). Figures 6c and 6d show, respectively, the sunspot number, *R*, and the number of sunspot groups, *N*_G_. In both cases, the data have been divided by the solar hemisphere, with blue for the north and red for the south. Both show clearly that sunspot activity dominated in the northern hemisphere initially in the cycle (being larger by a factor of up to 2) and that in the southern hemisphere dominated later in the cycle (again being larger by a factor of up to 2). This behavior was noted by *Waldmeier* [1957] and is not uncommon, in that the tendency can be seen for solar cycles 12, 14, 15, and 20–23 [e.g., *Zolotova and Ponyavin*, 2006; *Li et al.,* 2009; *Lockwood et al.,* 2012]. However, in none of these cycles is this feature as pronounced as it was for solar cycle 24.

**Figure 7:**
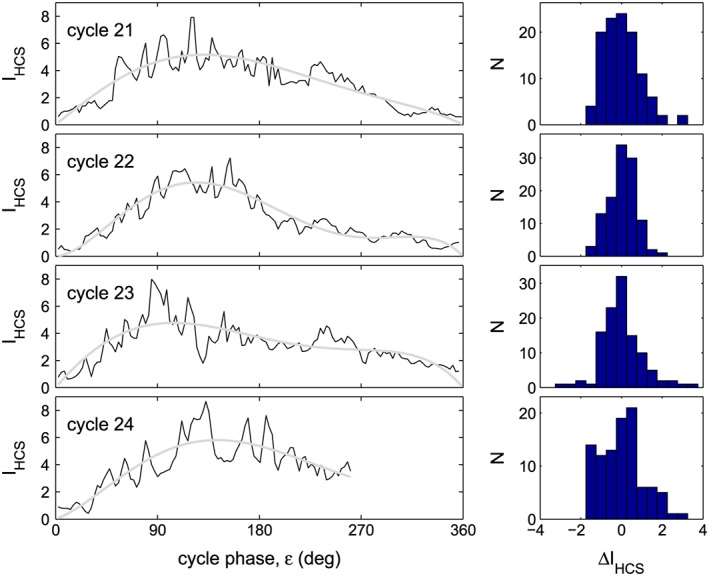
(left column) The variations of the current sheet tilt index *I*_HCS_ with solar cycle phase, *ε* for (from top to bottom) solar cycles 21–24. In each case the black lines are values for individual Carrington rotations and the grey line is the smoothed fifth‐order polynomial fit to these values [*I*_HCS_]_*s*_. (right column) The distributions of the deviations from the smoothed variation, Δ*I*_HCS_ = *I*_HCS_ − [*I*_HCS_]_*s*_ for solar cycle phase range 0 ≤ *ε* ≤ 270° (the range for which data are available for cycle 24 the time of writing). The values of *ε* are derived for each Carrington rotation from the mean latitude of observed sunspot groups using the procedure of *Owens et al*. [2011b].

Figure 7 demonstrates why we consider the fluctuations in *I*_HCS_ to have been relatively pronounced during cycle 24, by comparing with the corresponding data available for previous cycles. The left‐hand panels show the variations of the current sheet tilt index *I*_HCS_ as a function of solar cycle phase, *ε*. In each panel, the black lines are values for individual Carrington rotations and the grey line is the smoothed fifth‐order polynomial fit to these values [*I*_HCS_]_*s*._ The panels are (from top to bottom) for solar cycles 21–24. The values of the cycle phase *ε* are derived from the smoothed variation of the mean latitude of observed sunspot groups, using the procedure of *Owens et al.* [2011b]. All cycles show considerable fluctuations about the smoothed curves. The right‐hand panels show the corresponding distributions of the deviations from the smoothed variation, Δ*I*_HCS_ = *I*_HCS_ − [*I*_HCS_]_*s*_ for solar cycle phase range 0 ≤ *ε* ≤ 270° (the range for which data are available for cycle 24 at the time of writing). The distribution is somewhat broader for cycle 24 than for previous cycles, but other differences can also be seen in the variations with *ε*. Cycle 21 shows peak‐to‐peak variations in *I*_HCS_ that are almost as large as during cycle 24, but they are of higher frequency and so shorter lived. Cycle 22 shows smaller amplitude variations. Cycle 23 shows large amplitude and long‐lived fluctuations but only one positive one and one negative one. What we note about cycle 24 is that the fluctuations have been large, long lived, and persistent. It is this combination of characteristics that has helped us search for potential effects of *I*_HCS_ during cycle 24.

In Figure 6, a number of periods (labelled 1–5) have been defined by the vertical black lines. In Period 1 the northern hemisphere dominates the sunspot activity (as seen in Figures 6c and 6d); during Period 2 the two are more equal and oscillate around each other; Period 3 is when the dominant sunspot activity is in the southern hemisphere; this is true, to a lesser extent in Period 4; and in Period 5 the sunspot activity is very similar in the two hemispheres and the two decay together. In Period 4 the OSF rises steeply, as shown in Figure 6a. Note that the precise dates of the ends of Periods 4 and 5 were set by the ends of the available MErcury Surface, Space ENvironment, GEochemistry, and Ranging (MESSENGER) and HCS tilt data, respectively, whereas all other intervals were defined from the sunspot variations.

In Figure 6a the kinematic correction has been applied to the radial field data to allow for the effect of structure in the solar wind flow speed on the frozen‐in field which results in folds in the field lines and hence the modulus of the radial field |*B*_*x*_| increasing with heliocentric distance *r*, relative to a *r* ^−2^ dependence [*Owens et al.,* 2008]. A method to quantify this effect has been presented by *Lockwood et al.* [2009a, 2009c] and *Lockwood and Owens* [2009]. In Figure 6a, the dashed line gives the near‐Earth values of 2*π*|*B*_*x*_|(*r*_1_/*r*_*s*_)^2^ where *r*_1_ is an astronomical unit (1 AU) and *r*_*s*_ is the radius of the coronal source surface (here we adopt *r*_*s*_ = 2.5 *R*_**⊙**_, where *R*_⊙_ is a mean solar radius). This would be the (signed) OSF if an *r* ^−2^ dependence applied but exceeds the true (signed) OSF because at *r* = *r*_1_ there is folded flux which does not exist at *r* = *r*_*s*_, and so this means that there are field lines that thread the sphere or radius *r*_1_ multiple times yet only thread the source surface (at *r* = *r*_*s*_) just once, the difference being termed the “excess flux.” The solid line is the OSF with this excess flux removed, computed from the variation of the solar wind speed observed at *r* = *r*_1_ using the procedure developed by *Lockwood et al.* [2009a, 2009c]. The mauve line is this kinematically corrected OSF computed in the same way from the |*B*_*x*_| values measured by the MESSENGER spacecraft in orbit around Mercury. Because continuous solar wind speed measurements are not available from MESSENGER, the correction is based on the one made near Earth but scaled to the *r* of MESSENGER. Figure 6a shows that the kinematically corrected OSF values from near Earth and from near Mercury are very well matched.

**Figure 8:**
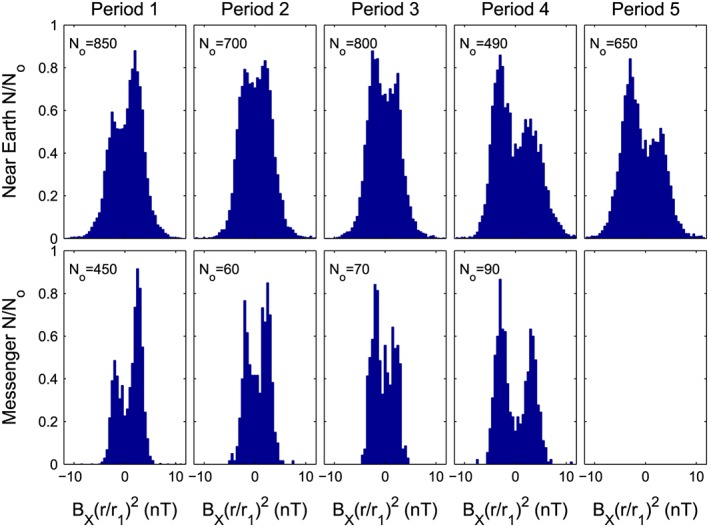
Distributions of the toward‐Sun field seen (top row) near Earth and at (bottom row) MESSENGER during selected intervals during solar cycle 24. The *B*_*X*_ component is positive toward the Sun and has here been multiplied by (*r*/*r*_1_)^2^ where *r* is the heliocentric distance of the point of observation and *r*_1_ = 1 AU. The MESSENGER data were taken between *r* = 0.31*r*_1_ and *r* = 0.47*r*_1_ and at heliographic latitudes between Λ_H_ = −3.4° and Λ_H_ = +3.4° (where Λ_H_ > 0 is in the northern solar hemisphere). For MESSENGER observations, all data used were for outside Mercury's magnetosphere and magnetosheath and sufficiently far upstream that no waves from the bow shock are detected. The near‐Earth data are continuous and from the OMNI2 data set: they are recorded between *r* = 0.98*r*_1_ and *r* = 1.02*r*_1_ and at heliographic latitudes between Λ_H_ = −7.2° and Λ_H_ = +7.2°. In each panel the number of samples in each 1 nT wide bin, *N*, has been divided by a scaling factor *N*_o_ which is given in each case.

Figure 8 shows the distributions of hourly means of the component of the magnetic field pointing toward the sun, *B*_*X*_, as seen near Earth (top row) and by the MESSENGER spacecraft in orbit around Mercury (bottom row). In both cases, the values have been approximately normalized to a heliocentric distance *r* = *r*_1_ by assuming an *r* ^−2^ dependence (i.e., distributions of *B*_*x*_ (*r*/*r*_1_)^2^ are shown). The MESSENGER data were taken between *r* = 0.31*r*_1_ and *r* = 0.47*r*_1_ and at heliographic latitudes between Λ_H_ = −3.4° and Λ_H_ = +3.4° (where Λ_H_ > 0 is in the northern solar hemisphere). For these distributions, only data recorded when MESSENGER was outside Mercury's magnetosphere and magnetosheath were used [*Winslow et al.,* 2013]. Furthermore, to avoid perturbations due to upstream waves from the bow shock, data from within 1 h of the bow shock intersection were also discarded. The near‐Earth data are continuous and from the OMNI2 data set: they are recorded between *r* = 0.98*r*_1_ and *r* = 1.02*r*_1_ and at heliographic latitudes between Λ_H_ = −7.2° and Λ_H_ = +7.2°.

The distributions are shown for each of the five intervals defined in Figure 6. During Period 1 flux emergence, as quantified by sunspot number *R* or the sunspot group number *N*_G_, was up to twice as large in the northern hemisphere as in the south. This means that there would be an excess OSF generated in the northern hemisphere, and, as Period 1 was during the rising phase of the solar cycle, sequence A of Figure 3 applies to this excess. Parts (c) and (d) of Figure 3 show that we expect there to be sunward flux near the solar equator in this case caused by the excess in northern hemisphere emergence. This can clearly be seen for Period 1 in Figure 8 as a dominance of *B*_*X*_ > 0 detected both at Earth and at MESSENGER. Note that Period 1 is 9800 hours long: the near‐Earth data coverage is 100% and the number of available hourly mean samples from MESSENGER is 3185 (giving a coverage of 32.5%). Because Period 1 (like Periods 2, 3, and 5) is slightly over 1 year long, it contains data over a full cycle of the Earth's Λ_H_ variation and over 4 cycles of Mercury's Λ_H_ variation. Period 4 is shorter and covers 0.78 of Earth's annual Λ_H_ variation and 3.2 cycles of Mercury's. Any systematic variations of *B*_X_ with Λ_H_ would be convolved with temporal variations by the orbital motions of the two planets and so very hard to detect. Nevertheless, tests were attempted by subdividing the distributions shown in Figure 8 into various Λ_H_ ranges (which gives reduced numbers of samples in each, which is a particular problem for the MESSENGER data) but no systematic variations with Λ_H_ could be detected.

In Period 2, the sunspot numbers were more equal in the two hemispheres and the north/south ratio for both *R* and *N*_*G*_ oscillated around unity. In this interval, the northern polar field has flipped but the southern field has not (discussed below and shown in Figures 10b and 10d, respectively) and so either Figure 1a or Figure 2a applies in the southern hemisphere, but either Figure 1b or Figure 2b applies in the north. The relevant parts of Figures 1 and 2 predict both polarities of *B*_*X*_ at both Earth and MESSENGER in this case. Figure 8 shows that this is indeed observed for Period 2. There remains a slight dominance of *B*_X_ > 0 at both locations in this interval, which we could be attributed to time lags, for example, between emergence through the solar surface (giving sunspots) and emergence through the coronal source surface and the time required for flux to either migrate away from the equator or be disconnected by HCS reconnection. The near‐Earth data for Period 2 has 9208 out of a possible 9216 hourly mean samples (99.9% coverage) whereas the MESSENGER data has just 471 (5.1% coverage).

For Period 3, both polar field flips have largely completed and sunspots in the southern hemisphere dominate. Thus, Figure 4b applies. Parts (h) and (i) of Figure 4b predict predominant away field (*B*_X_ < 0), and this can be seen in Figure 8 for Period 3 at both Earth and Mercury. Period 3 has 100% coverage for near‐Earth data, whereas MESSENGER gave 549 samples out of a possible 10,104 (5.4% coverage). We would expect the same behavior to apply for Period 4. Figure 8 shows that this is true for both the near‐Earth and the MESSENGER data. In this interval, the near Earth data has 100% coverage of the 6792 hours whereas MESSENGER gave 786 hours (11.6%). Lastly for Period 5 the near‐Earth data show that the dominance of *B*_X_ < 0 data persists. These data have 100% coverage of the 8118 hours. Period 5 is after the end of the MESSENGER mission.

We need to treat the distributions for MESSENGER with caution because of the sampling. The low orbital period of Mercury means that the mean solar rotation period, as seen from there, is 34.8 days and observations from Earth indicate an approximately two‐sector structure of the IMF for most of the declining phase of cycle 24 so MESSENGER then spends typically 15 days in each sector. There are data gaps in this data set and some are as long as 15–30 days. Gaps lasting 30 days do not bias the results that much as they cover approximately a full solar rotation, as seen from Mercury. However, the 15 day gaps are a problem as, depending on when they occur, they have the potential to affect predominantly one sector and so change the shape of the *B*_*X*_ distribution. However, this problem is not a factor for the distributions for the near‐continuous near‐Earth data, and it is interesting to note that the MESSENGER data do reflect the asymmetries in the occurrence of toward and away interplanetary magnetic field that are seen in the near‐Earth data.

Subsequent to the present paper being submitted, *Dósa and Erdős* [2017] have published a paper which makes a different comparison between the MESSENGER and near‐Earth heliospheric field data. These authors show that there is longitudinal structure in the OSF with marked peaks at Carrington longitudes around 210° and 330° (their Figure 8). Their data were compiled over the year 2008 (Carrington rotations 2066–2078). In this time the source surface maps generated using PFSS by the Wilcox Solar Observatory (http://wso.stanford.edu/synsourcel.html) show a classic tilted main HCS, with clear peak excursions in heliographic latitude. We here note that during 2008, the peak HCS excursion to the north of the heliographic equator evolves from Carrington longitude 175° to 220° and that to the south evolves from 272° to 338°. Hence, the enhanced OSF is at exactly the longitudes predicted by *Owens et al.* [2011a] who argued that OSF disconnection occurs where the HCS is tilted relative to the direction of rotational motion (i.e., where oppositely directed OSF is brought into contact by solar rotation) and not at the peaks of the HCS excursions where the HCS is not inclined with respect to the direction of solar rotation. *Dósa and Erdős* [2017] show that compression by fast flows is a factor but our analysis shows some effect remains when this is allowed for, potentially indicating the effect of HCS tilt on OSF disconnection predicted by *Owens et al.* [2011a].

## 4 Solar Cycle 24 and Magnetograph Observations of the Photospheric Field

**Figure 9:**
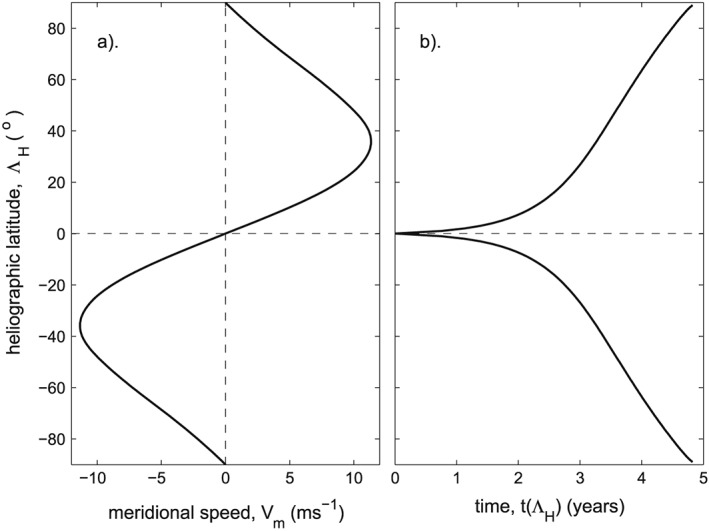
Meridional motion of photospheric magnetic features. (a) The poleward meridional velocity as a function of heliographic latitude, Λ_H_, as derived by *Hathaway and Rightmire* [2011]. (b) The time taken to reach latitude Λ_H_ from very close to the heliospheric equator (|Λ_H_| = 0.5°).

In this section, we study the poleward propagation of magnetic features of the photosphere seen in the magnetograms generated by magnetographs. To understand the motion expected, we here use the profile of meridional velocities *V*_*m*_ derived by *Hathaway and Rightmire* [2011], shown in Figure 9a. This is derived from observations of the progression of magnetic structures and agrees closely with the profiles used by *van Ballegooijen et al.* [1998] and by *Schrijver and Title* [2001]. It also agrees quite well with the profiles derived from Doppler shifts [e.g., *Ulrich*, 2010] and, where comparisons are possible, with the results from helioseismology [e.g., *Zhao and Kosovichev*, 2004; *Švanda et al.,* 2007]. In Figure 9b, this profile has been converted into the time *t*(Λ_H_) for magnetic flux to reach a given latitude Λ_H_ from a starting point very close to the solar equator (|ΛH| = 0.5°). Figure 9b is used to predict trajectories on the magnetograms that originate at a general initial latitude Λ_H0_ because the time to reach a greater latitude, Λ_H_, is *t*(Λ_H_) − *t*(Λ_H0_).

Note that one model flow profile, which is used by of *Wang et al.* [2009], is considerably different from that in Figure 9a because it gives meridional flows that stay at large magnitudes at Λ_H_ almost down to the solar equator and then decreases very rapidly with decreasing |Λ_H_|, generating close to an equatorial discontinuity in the meridional speed profile *V*_*m*_(Λ_H_). This means that the *Wang et al.* [2009] model profile would not predict the much longer evolution times to move away from the solar equator (compared to those for greater |Λ_H_|) that can be seen in Figure 9b. Hence, the trajectories on magnetograms predicted by *Wang et al.* [2009], while very similar to those used here at latitudes above about 30°, are considerably different at equatorial latitudes. We adopt the *Hathaway and Rightmire* [2011] profile because it agrees closely in form with the Doppler measurements [*Ulrich*, 2010] in showing more gradual gradient in *V*_*m*_ across the solar equator. The significance of the difference between the *Wang et al.* [2009] profile and the others (including that by *Hathaway and Rightmire* [2011] used here) is that the others give significantly longer transit times from the point of emergence (at latitude Λ_H0_) to near the pole for later in the solar cycle when Λ_H0_ of emerged flux is lower.

Before discussing the magnetograms, we need to clarify our approach to their interpretation in this paper. Magnetographs measure the total flux in the photosphere, i.e., both closed loops and open flux. OSF accounts for about 4% of the total photospheric flux at sunspot maximum, rising to about 40% at sunspot minimum. The HCS tilt mechanism that we discuss only relates to the disconnection of OSF. The OSF has foot points that are embedded in the photosphere and which move both under the general pattern of photospheric motions, and also because interchange reconnections cause them to jump from one point to another. In the active region belts, the OSF is a small fraction of the total; however, as photospheric flux migrates poleward, OSF also tends to evolve poleward and, in addition, the total closed flux decreases greatly at the poleward edge of the active region belt such that a larger fraction of the total flux is open with increasing latitude (until it reaches the polar coronal hole when almost all the flux is OSF). Any HCS tilt effect on the OSF will therefore become increasingly more apparent as the foot points evolve poleward.

### 4.1 Zonally Averaged Magnetograms

**Figure 10:**
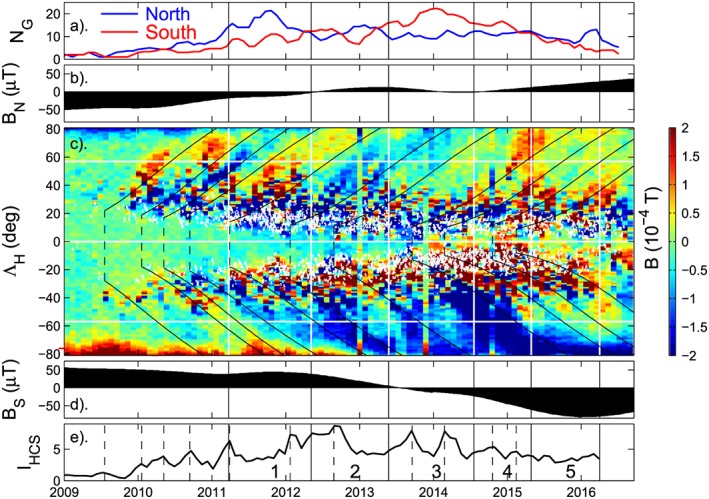
Photospheric field evolution during solar cycle 24. (a) The northern (in blue) and southern (in red) hemisphere sunspot group numbers (as in Figure 5d); (b) the northern polar photospheric magnetic field strength, *B*_N_, from the WSO magnetograph data, passed through a 20 nHz low‐pass filter to remove the annual variation due to viewing geometry; (c) the zonal (longitudinal) mean of the photospheric field from the Global Oscillation Network Group (GONG) magnetographs, as a function of heliographic latitude (Λ_H_) and time. The white dots are the locations of the centers of sunspot groups detected by the Solar Observing Optical Network (SOON), and the black lines are predicted propagation profiles (from Figure 9b) for events starting from the predicted mean latitude of spots of “leading polarity” magnetic field at the times marked by vertical dashed lines in Figure 10e, when there are peaks in *I*_HCS_. (d) The southern polar photospheric magnetic field strength, *B*_S_, from the WSO magnetograph data, passed through the same 20 nHz low‐pass filter as *B*_N_. (e) The heliospheric current sheet tilt index, *I*_HCS_, as shown in Figure 5b. The vertical solid lines in all panels define the intervals 1–5 and the horizontal white lines in 10c are at Λ_H_ ± 57°, as used in Figure 9.

Figure 10c presents a magnetogram for solar cycle 24 thus far (a map of zonally averaged radial magnetic field in a frame of heliographic latitude, Λ_H_, and time) compiled from the Global Oscillation Network Group (GONG) magnetograph data. No smoothing has been applied to the pixels (which are 1° in Λ_H_ and one Carrington rotation period in time) and the color scale saturates at ±2 × 10^−4^ T. Λ_H_ is positive toward north, and red (blue) denotes positive (negative) field which is away (toward) the Sun. (Note that this is the opposite sign convention used for the near‐Earth and near‐Mercury interplanetary data in Figure 8 for which *B*_*X*_ > 0 is toward the Sun) The white dots show the locations of centers of sunspot groups detected by the Solar Observing Optical Network (SOON) and reveal the well‐known “butterfly” pattern of emerged photospheric flux in bands in both hemispheres which migrate toward the equator as the cycle evolves. To the poleward side of these active region bands are seen what are commonly referred to as “poleward surges.” This term is somewhat misleading because it implies increases in the speed of poleward motion, whereas the features on the magnetograms tend to be noticeably parallel at each Λ_H_, showing a rather steady and constant poleward motion profile. In other words, the meridional circulation provides a relatively steady “conveyor belt” which can carry fields of varying strength and even of opposite polarities. Indeed, the strongest features that can be seen in Figure 10c are caused by the zonal mean of the field in one hemisphere changing polarity from inward to outward or vice versa. The black lines are predicted trajectories of field lines and are discussed below.

Figures 10b and 10d show the magnitude of the average fields over the northern and southern solar poles (*B*_N_ and *B*_S_, respectively) from the Wilcox Solar Observatory (WSO) magnetograph [*Scherrer et al.,* 1977]. These data have been passed through a 20 nHz low‐pass filter to remove the annual oscillation caused by the annual variation in Λ_H_ of the ground‐based magnetograph that made the observations. It can be seen that the polar field in the southern hemisphere, *B*_S_ (Figure 10d), flipped polarity close to the end of Period 2, and this is relatively late compared to other solar cycles (see *Lockwood* [2013]—also discussed later in relation to Figure 13 of the current paper). Comparing Figures 10b and 10d shows that this is caused by the arrival a large amount of negative (inward) magnetic flux (colored blue) at the southern pole. On the other hand, the northern polar field *B*_N_ flipped polarity close to the end of Period 1, and this is relatively early compared to previous cycles. Unusually, this northward field then reversed again before finally acquiring the new polarity at the end of Period 3. This sequence can be seen to be consistent with the average polarity of the poleward moving flux in the northern hemisphere, with field that oscillates (in zonal averages) between outward (red) and inward (blue). In particular, the temporary change back to negative *B*_N_ is the effect of poleward migration of the inward flux (blue region), as pointed out by *Sun et al.* [2015] and *Mordvinov et al.* [2016].

For comparison, Figures 10a and 10e show the sunspot group number *N*_G_, and the HCS tilt index *I*_HCS_ as in Figures 6d and 6b, respectively. The vertical black lines in all panels (white in Figure 10c) delineate the five periods, as in Figure 6.

The dashed vertical lines in Figure 10e mark a series of peaks in *I*_HCS_. Also shown in Figure 10c are a series of black lines which are the expected loci of magnetic features in the photosphere, predicted using Figure 9b. These start from an initial heliographic latitude Λ_H0_ which is the mean latitude of the sunspots in that hemisphere for the Carrington rotation period of the time of the *I*_HCS_ peak, minus a small offset of *δ*Λ. This offset is included because, for reasons discussed in section 5, we are particularly interested in the potential poleward evolution of the leading spot field polarity and such spots originate, on average, at lower latitude than the trailing ones (because of Joy's law). We here adopt a value for *δ*Λ between the mean latitude of all spots and the mean latitude of the leading spots from the formula of *Wang and Sheeley* [1991]:

(1) $$\delta \Lambda \approx 5\;\tan\left(\Lambda_{H}/2\right).$$

For Λ_H_ of 30° (typical of just after the start of a solar cycle), this yields *δ*Λ ≈ 1.4°, and for Λ_H_ of 5° (typical of just before the end of a solar cycle), it yields *δ*Λ ≈ 0.2°.

### 4.2 Longitudinal Structure at Fixed Latitude

**Figure 11:**
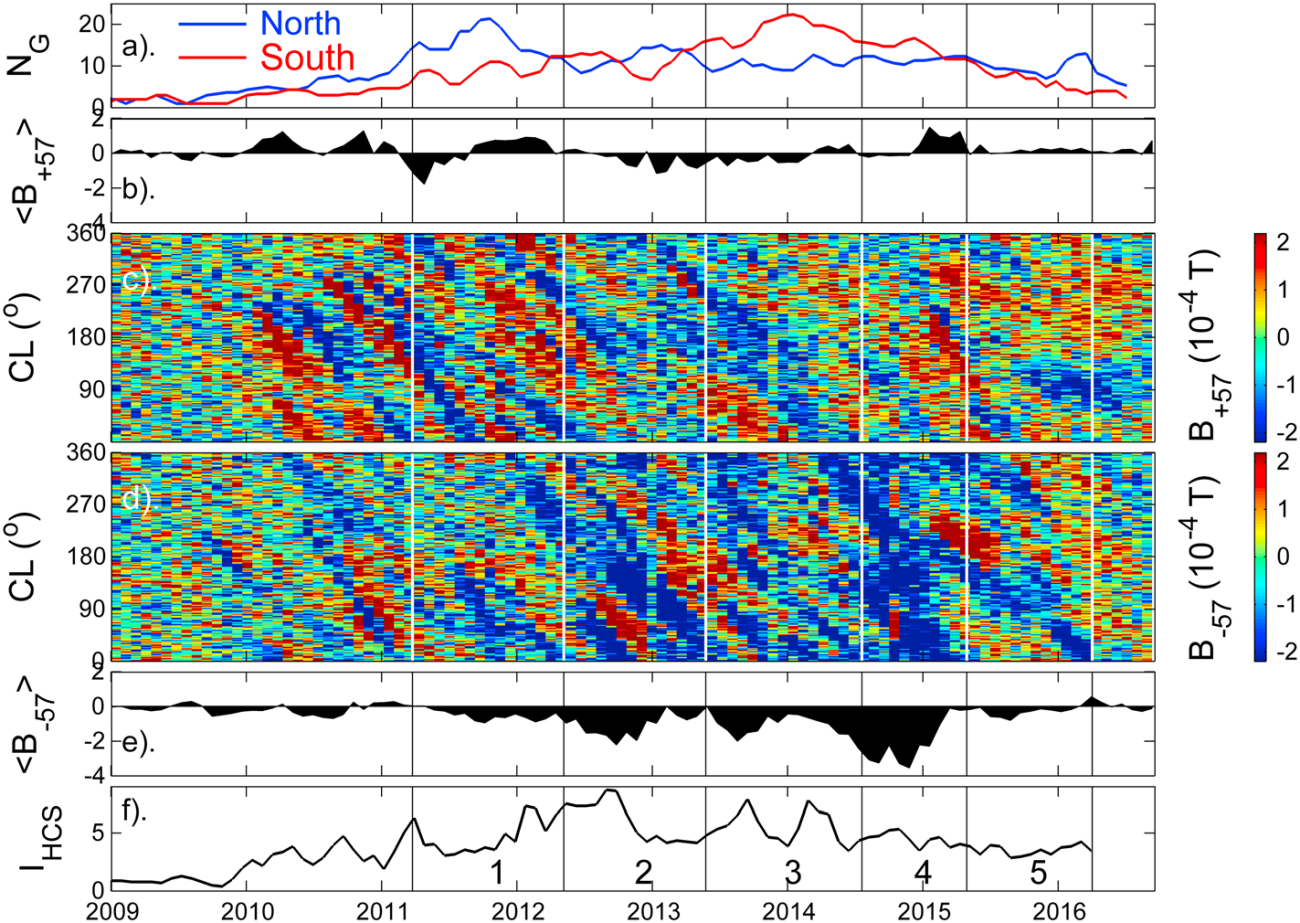
Longitudinal slices of the photospheric field from the GONG magnetograms at heliographic latitudes of (c) Λ_H_ = +57°, *B*_+57_, and (d) Λ_H_ = −57°, *B*_−57_. (b and e) The zonal mean values as a function of time, <*B*_+57_ > and <*B*_−57_ > are shown. (a and f) The hemispheric sunspot group numbers *N*_G_ and the HCS tilt index, *I*_HCS_, as in the top and bottom rows of Figure 8. The vertical lines delineate the five intervals studied.

A point to be remembered about Figure 10c and similar magnetograms discussed in other papers [e.g., *Mordvinov et al.,* 2016; *Yeates et al.,* 2015; *Sun et al.,* 2015; *Petrie et al.,* 2014] is that the fields shown are zonal means and so they show the net result of all structures at that latitude (i.e., at all longitudes). Because of the dependence of HCS disconnection of OSF on the HCS tilt angle, we expect that different longitudes may behave differently at any one time: specifically at longitudes where the HCS is close to the equator and more highly tilted, we expect OSF disconnection at the HCS to occur (and hence a schematic like Figure 1 applies) whereas at other longitudes where the HCS is at higher latitudes and less tilted HCS disconnection of OSF is less likely and a schematic like Figure 2 would apply. Hence, it is important to look at the longitudinal structure, and Figures 11c and 11d show longitudinal slices, of the kind used by *Bumba and Howard* [1965], at Λ_H_ = +57° and Λ_H_ = −57°, respectively (these latitudes are shown by the horizontal lines in Figure 10c, chosen as they are approximately the lowest |Λ_H_| that remains above the sunspot latitudes throughout the solar cycle. Results are similar for any other latitude above 57°. The field is for pixels that are 1° in Λ_H_ by 1° in Carrington longitude (CL). Figures 11b and 11e show the zonal means, <*B*_+57_> and <*B*_−57_>, as a function of time are so are the time slices along the horizontal white lines in Figure 10c. As for Figure 10, Figures 11a and 11f show the sunspot group number *N*_G_, and the HCS tilt index *I*_HCS_ (as in Figures 6d and 6b) for reference and the vertical black lines (white in Figures 10b and 10c) delineate the five periods, as in Figure 6.

The most obvious features in Figures 11c and 11d are the inclined regions of field, of both polarities, that migrate to lower CL with time. This is because regions are rotating more slowly at Λ_H_ = +57° and Λ_H_ = −57° than the center of the sunspot belt, and the latter sets the Carrington rotation period and hence the CL. The key point is that at most times, both field polarities were seen moving poleward at some longitude in both hemispheres, a fact that is hidden in the zonal means shown in the commonly used latitude‐time magnetograms (such as Figure 10c).

In the rising phase of cycle 24 emergence was dominant in the northern hemisphere and Figure 11c shows that it caused a series of patches of positive (away, in red) field that is usually followed at the same CL by a period of negative field (toward, in blue). There are two clear examples of this that start before Period 1. This is consistent with Figure 3a which predicts that negative field will, after some delay, follow the positive field poleward if disconnection at the HCS cannot keep pace with the northern hemisphere emergence. There is, however, an interesting difference in the middle of Period 1 when the region of negative flux is much shorter lived. This could well relate to the brief spike of enhanced HCS tilt index at the start of Period 1 which could act to enhance the HCS disconnection rate. The relative absence of the negative field would be delayed because it takes time to propagate in latitude up to Λ_H_ = +57°. Note that in the northern hemisphere, during the interval before the middle of 2012 only once (at the start of period 1) was there enough negative flux to make the zonal mean turn negative (Figure 11b) but that negative flux was present at some longitude all the time. This is not the case for Periods 2 and 3 when <*B*_+57_> is consistently negative despite some poleward moving patches of *B*_+57_ > 0 (in red in Figure 11c). As demonstrated by Figure 10, this is the magnetic field that temporarily reversed the flip in the northern polar field. Figure 11c shows that for intervals 4 and 5, and after, more longitudes were seeing poleward motion of positive flux in the northern hemisphere and <*B*_+57_> was generally positive or near zero.

Figure 11d shows that in the southern hemisphere during Period 1 there were three, relatively weak, examples of patches of negative flux followed by positive, implying that even for the relatively weak southern hemisphere emergence, it was not all reconnected at the HCS, i.e., Figures 2a and/or 5a were relevant to some extent as well as Figures 3a and 1a. However, for Periods 2, 3, and 4 there was a strong dominance of poleward moving negative flux, which was reflected in the strongly negative <*B*_−57_> in Figure 11e. However, positive flux is still always seen after an increase in negative and acts to reduce the magnitude <*B*_−57_> at any one time but is not sufficient to reverse its polarity. By the end of Period 5 only positive field is seen moving poleward at all longitudes briefly giving <*B*_−57_> > 0. This appears to be associated with the slight reduction the negative average southern polar field *B*_S_ at this time, as can be seen in Figure 9d.

### 4.3 Hemispheric Differences and Total Flux Transport

**Figure 12:**
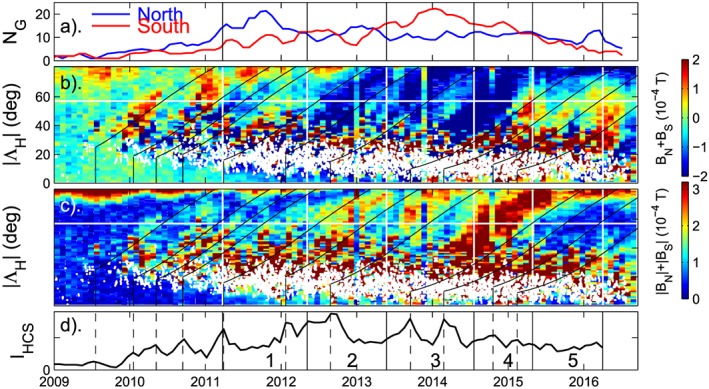
Hemispheric comparisons of zonal (longitudinal) means of the photospheric field from the GONG magnetographs. (b) The sum of the zonal means in the fields in the northern and southern hemisphere at the same magnitude of heliographic latitude, |Λ_H_ |, *B*_N_(|Λ_H_ |) + *B*_S_(|Λ_H_ |), as a function of |Λ_H_|. (c) The sum of the moduli of the zonal means in the fields in the northern and southern hemisphere at the same magnitude of heliographic latitude, |Λ_H_ |, |*B*_N_(|Λ_H_|)| + |*B*_S_(|Λ_H_|)|, as a function of |Λ_H_|. In Figures 11b and 11c the white dots show the |Λ_H_| of sunspot group centers in the SOON data (from both hemispheres). The trajectories start from the predicted mean latitude of “leading polarity” emerged magnetic field |Λ_H0_ | for both hemispheres at the times of *I*_HCS_ peaks, marked by the vertical dashed lines in Figure 11d. (a and d) The sunspot group numbers *N*_G_ and the HCS tilt index, *I*_HCS_—as in the top and bottom rows of Figure 8. The vertical lines delineate the five intervals studied.

Figure 12 presents an analysis of the hemispheric asymmetries and the global poleward transport of photospheric flux. Figure 12b presents, in a time‐|Λ_H_| format, the sum of the zonal‐mean field in the two hemispheres at the same latitude, *B*_N_(|Λ_H_|) + *B*_S_(|Λ_H_|). For cycle 24, the expected dominant flux transport is positive in the northern hemisphere and negative in the south; hence, *B*_N_(|Λ_H_|) + *B*_S_(|Λ_H_|) is expected to be positive when the emergence in the northern hemisphere dominates and negative when that in the southern hemisphere dominates. Comparison of Figures 12a and 12b indicates that this was generally the case. Period 2 is interesting as *B*_N_(|Λ_H_|) + *B*_S_(|Λ_H_|) emerging from the sunspot belt is strongly negative even though sunspot numbers are roughly equal in the two hemispheres. Inspection of Figure 11d shows that at this time negative flux is propagating poleward in both hemispheres, suggesting that it is the expected high‐latitude trailing (negative) field polarity in the southern hemisphere, but the leading, lower latitude (negative) field in the north generated by the earlier dominance in northern sunspot numbers and which has escaped reconnection in the HCS. This situation can arise because of the large increase in propagation time with decreasing |Λ_H_| that is predicted by Figure 9b. This agrees with the slight dominance of sunward field at Earth and Mercury that persists into this period (Figure 8b).

Figure 12c sums the flux of either polarity in either hemisphere heading toward the poles, |*B*_N_(|Λ_H_|)| + |*B*_S_(|Λ_H_|)|. From Figures 1, 2, 3, 4, 5 the only way that this can be reduced, for a given total emergence in both hemispheres, is by an increase in the reconnection in the HCS (the lost flux being removed along the streamer belt). In Figure 12c the times of peak *I*_HCS_ are again marked by vertical dashed lines and evolution locus for each peak is shown by a black line starting from initial heliographic latitude Λ_H0_ which is the estimated mean latitude of the leading sunspots in both hemispheres at that time. Inspection of Figure 12c shows that the total poleward motion of magnetic flux of both polarities is decreased around the time of each black locus line. This is the an indication that increased HCS tilt angle increases the flux disconnection rate at the HCS, as postulated theoretically for OSF by *Owens et al.* [2011a] and on the basis of white light coronagraph observations by *Sheeley and Wang* [2001] and *Sheeley et al.* [2001].

## 5 Discussion and Conclusions

### 5.1 The Origin of OSF That Progresses the Solar Cycle in Polar Fields

In their model of the flux transport that leads to the solar cycle evolution of polar fields, *Zolotova and Ponyavin* [2012] argued that bursts of rapid emergence of flux through the photosphere lead to a poleward surge in the flux of the trailing spot field polarity moving toward the pole and that, between these bursts, field of the polarity of the leading spots drifts poleward. These authors ascribed the fact that there is a “surplus” of the trailing spot polarity field arriving at the poles (which advances the solar cycle evolution of the polar field) to a surplus in the emerged flux of the trailing polarity. In fact, because emerged flux comes in loops from the toroidal field stored in the overshoot layer at the base of the convection zone, the leading and training flux polarities must be equal in total. (Note that either the leading polarity or the trailing polarity flux could be more dispersed into faculae than the other than giving an excess in sunspot flux but this will not be present in the total emerged flux). From the results presented here a different picture emerges whereby the “surplus” is generated in two ways—either by reconnection of leading spot polarity field in the heliospheric current sheet, as proposed by *Lockwood* [2004] and illustrated in Figure 1, or by diffusive motion of leading spot polarity field lines across the equator [*Petrovay*, 2010; *Cameron et al.,* 2013], as illustrated in Figure 5.

As pointed out by *Sun et al.* [2015], the zonal mean magnetographs in Figure 10 do indeed show that the average poleward flux has the trailing spot polarity and poleward “surges” follow a burst of sunspot activity in that hemisphere and that, during lulls between such activity, the average of the poleward moving field can take on the polarity of the leading spots. However, these zonal means do not tell the whole story because, as Figure 11 demonstrates, both polarities are generally moving poleward at all times and that some leading spot polarity field is migrating poleward at longitudes away from the active regions.

Figure 12b is the most revealing when it comes to studying the role of field line disconnection at the heliospheric current sheet as it shows the total flux of either polarity moving poleward in both hemispheres, |*B*_N_(|Λ_H_|)| + |*B*_S_(|Λ_H_|)|. The black lines show the trajectories of field lines from the expected location of the leading spots at times when the HCS tilt index shows a peak. It can be seen that this total flux of field lines is reduced along each one of these trajectories. It is instructive to consider what caused these decreases in the total poleward flux transport in the photosphere. All the sequences in Figures 1, 2, 3, 4, 5 will give such a decrease if the rate of flux emergence is decreased. However, neither the sunspot number *R* nor the sunspot group number *N*_G_ (Figures 6c and 6d) show any evidence of decreases at the required times. The important point is that for the mechanisms shown in Figures 2–5, decreases in |*B*_N_(|Λ_H_|)| + |*B*_S_(|Λ_H_|)| could only arise from decreases in emergence rate. The sequence illustrated in Figure 1 is different as in that it can give a decrease in |*B*_N_(|Λ_H_|)| + |*B*_S_(|Λ_H_|)| from a drop in emergence rate or from a rise in the HCS reconnection rate. Given the lack of any evidence for the former in the sunspot data, the decreases seem to arise from the latter.

Hence, our explanation for these drops in the total poleward flux of field of either polarity is that the leading spot field polarity is reduced each time the current sheet index shows a maximum, removed by disconnection via reconnection in the HCS. This has been discussed previously in terms of OSF loss, but Figure 12 suggests it is also converting pairs of closed loops in the two hemispheres into single loops with foot points in opposing hemispheres and of trailing spot polarity. This causes the subsequent total migration of magnetic flux (of both polarities) to be reduced, as in Figure 1, compared to that when the HCS reconnection is absent or at a lower rate, as in Figure 2. Hence, Figure 12 provides the first direct evidence that the current sheet tilt increases the HCS disconnection rate, as proposed theoretically for OSF by *Owens et al.* [2011a] and on the basis of white light coronagraph observations by *Sheeley and Wang* [2001] and *Sheeley et al.* [2001]. Previously, the only other evidence for this effect was that long‐term reconstructions of OSF were improved if the OSF loss rate was given the solar cycle dependence of the HCS tilt [*Owens and Lockwood,* 2012]. Note that we call this “evidence for” rather than “proof of” the role of OSF disconnection at the HCS at this stage. Work is underway to quantify the decreases in |*B*_N_(|Λ_H_ |)| + |*B*_S_(|Λ_H_|)| by modeling, and if quantitative agreement between the two is good, we would regard that as a higher level of proof. Note that we are certainly not stating that the sequences illustrated in Figure 5 do not occur but we are providing evidence that those shown in Figure 1 do. Without quantification by modeling, we cannot say anything about the relative contributions of these two classes of sequence to the advancement of the solar cycle via their contribution to the polar field evolution.

The trajectories in Figure 12 are based on the mean latitude of leading spots (at the times of the peaks in HCS tilt) in both hemispheres. The trajectories in Figure 10 are based on the mean latitudes of spots for each solar hemisphere separately. These show that the leading spot polarity field migrates poleward from the lulls in HCS disconnection when the HCS tilt is lower: there is a clear tendency to see either a reduction in the average field of the trailing polarity or even field of the leading polarity in the gaps between the black trajectories shown. Good examples are for the last four pairs of trajectories shown. In the southern hemisphere we see leading spot polarity in these zonal means (in yellow or red) between the trajectories, and along the trajectories we see the trailing spot field polarity (in cyan or blue) in the zonal means. At the same time, in the northern hemisphere we see training spot polarity at all times in the zonal means but these average values are reduced between the trajectories (usually from orange/red to yellow/green on the color contours) which implies that more of the leading spot polarity is moving poleward because of the lulls in HCS reconnection. The reason why the leading spot polarity migration has a smaller effect on the zonal means in the north than in the south is because in Periods 3 and 4 southern hemisphere emergence dominated, meaning that there is more field of leading spot polarity at low latitudes in the southern hemisphere than in the north. This is confirmed by the near‐Earth and MESSENGER interplanetary data that see a predominance of away field (*B*_*X*_ < 0) in Periods 3, 4, and 5 (see Figure 8).

In interpreting the magnetograph data, the long delays in the effects at middle and high latitudes of increased and decreased HCS tilt are important. These are expected because HCS disconnection is fastest where the HCS tilt is greatest and that is at low heliographic latitudes. The latitudinal profile of meridional flow (Figure 9) makes the propagation times from low to middle and polar latitudes long.

### 5.2 Causes and Effects of Poleward Migration of Leading‐Spot Polarity Field

The tendency for the leading spot polarity field to follow the trailing one poleward following lulls of the OSF disconnection rate at the HCS can also be seen in the northern hemisphere, particularly between the second and third trajectories shown. This event has also been studied and modeled by *Yeates et al.* [2015]. Looking at the time series of zonal means at Λ_H_ = +57° in Figures 10c and 11b, this is seen as a bipolar signature of positive (away, red), followed by negative (toward, blue) field which is centered on the start time of Period 1. However, the longitudinal slices for Λ_H_ = +57° (Figure 11c) show that this is more complex than the zonal means imply in that both field polarities are seen in this event both before and after the zonal‐mean polarity reversal with away flux (in red) being followed at the same longitude (evolving from CL ≈ 270° to CL ≈ 120°) by toward flux (blue) and it is only the balance between the two that changes at the polarity reversal. Notice also that the zonal means are also complicated by a second event taking place at lower CL at the same time. The trajectories in Figure 10 show that the poleward migration of leading polarity flux (i.e., toward field in blue) comes from a prior lull in the HCS reconnection rate. This feature has also been interpreted and modeled as due to a large non‐Joy's law BMR [*Yeates et al.,* 2015]. This is not necessarily inconsistent with the ideas discussed here as the observed HCS tilt angle variation is likely to be have been strongly influenced by this his non‐Joy's law BMR. This means that using the HCS tilt angle to quantify OSF disconnection at the HCS (as used by *Owens and Lockwood* [2012]) and using sunspot BMR tilt angle to model its subsequent poleward evolution and cross equator drifts (as used by *Cameron et al*. [2014]) may be approaches that are more closely related than they previously appeared to be. Note that the same considerations apply to BMRs that do not obey Joy's law as to those that do, in that if both polarity foot points evolve to the same pole, the BMR does not advance the solar cycle. The non‐Joy's law tilt may, however, influence the probability of this occurring.

Lastly, we note that *Sun et al.* [2015] that find the trailing spot polarity field poleward surges tend to be associated with strong activity and BMRs that obey Joy's law whereas the leading spot polarity surges tend to be associated with lower activity intervals, as noted here. This is not inconsistent with what is proposed here. However, *Sun et al.* [2015] attribute the leading spot polarity surges to modulation of the meridional flow (due to field‐dependent convergence of flow toward the ARs), whereas we are attributing to a relative lack of transequatorial transport for closed flux and reduced HCS reconnection for OSF.

Figure 10 provides an explanation of the behavior of the polar fields shown in Figures 10b and 10d. As noted by *Sun et al.* [2015] and *Mordvinov et al.* [2016], the temporary reversal of the northern polar field is due to an extended period where leading spot polarity (toward in the northern hemisphere) reaches the northern polar coronal hole. This is seen in blue bands in the northern hemisphere of Figure 10c that again fall between the trajectories that map to the peaks in HCS tilt and arises in Period 1 when the northern hemisphere dominated emergence through the photosphere. This excess of undisconnected toward flux in the northern hemisphere (leading spot polarity) available to move poleward is consistent with Figure 3a and with the dominant *B*_*x*_ > 0 seen at Earth and MESSENGER during Period 1. Hence, the temporary reversal in the northern polar field was caused by the early dominance of northern hemisphere emergence, with an additional contribution of a lull in the HCS reconnection rate (which meant that the rate of disconnection with the lower flux of southern hemisphere leading polarity flux that had emerged was limited in rate).

### 5.3 The Rapid Rise in OSF During the Declining Phase of Cycle 24

The other general feature of interest is the large rise of OSF during Period 4 shown in Figure 6a. Figure 10c shows that this was due to strongly asymmetric emergence dominated by the southern hemisphere. Again, it formed during a prior lull in the OSF disconnection rate at the HCS (in Period 3), ensuring that the HCS disconnection rate was reduced, which would also be low because of the smaller amount of leading polarity flux generated in the northern hemisphere at this time. It has often been assumed that the rise in OSF during cycle 24 was due to enhanced OSF emergence, but as pointed out by *Wang and Sheeley* [2016a] and *Wang* [2016], no increase in coronal mass ejection rate was seen at this time, and CME rate has been a successful predictor of OSF production in some continuity models [*Owens and Lockwood*, 2012]. The data presented here offer an explanation in that it was a reduction in the loss rate that caused the OSF rise, rather than an increase in the production rate. That reduction has two causes: first the dominance of emergence in one hemisphere (the southern) meant that part of the leading polarity flux could not be disconnected by HCS reconnection and second, there was a lull in the HCS disconnection rate (caused by a reduction in the HCS tilt angle) which meant that the removal of flux that was matched in the two hemispheres was not as rapid as it otherwise would have been.

The analysis presented here offers an explanation of why the two‐peaked nature of the solar cycle (separated by the so‐called “Gnevyshev gap” [*Gnevyshev,* 1963; *Norton and Gallagher*, 2010]) is particularly clear in OSF [*Lockwood et al.,* 1999, 2009a] and galactic cosmic ray (GCR) fluxes [e.g., *Storini et al.,* 1997] and often is also asymmetric in the amplitude of the two peaks. Cycle 24 is an example where this gap is present in sunspot numbers and it is the northern hemisphere which dominated flux emergence before the Gnevyshev gap and it is the southern hemisphere that dominated after it. Both peaks in OSF (minima in GCR fluxes) are due to OSF emergence, but the second is amplified by the temporary accumulation of OSF at the poles, of both leading and trailing spot polarity, due to asymmetric emergence and reduced flux disconnection rates in the HCS.

### 5.4 Implications for the Remainder of Cycle 24 and for Cycle 25

The above analysis has an interesting implication. Following the large rise in OSF and the strong migration of southern hemisphere trailing polarity flux toward the southern pole in Period 4, there follows (toward the end of Period 4, and throughout and after Period 5) undisconnected leading polarity flux moving poleward in the southern hemisphere, especially in the lulls between HCS disconnection rate peaks. This can be seen to be having an effect on the southern polar field in Figure 10d which is, at the time of writing, falling and has been throughout 2016. This is consistent with the evolution of the temporary OSF enhancement predicted in Figure 4b for the enhanced emerged flux in the southern hemisphere. At the same time the northern hemisphere polar field is rising slowly due to the arrival of trailing polarity flux in that hemisphere.

**Figure 13:**
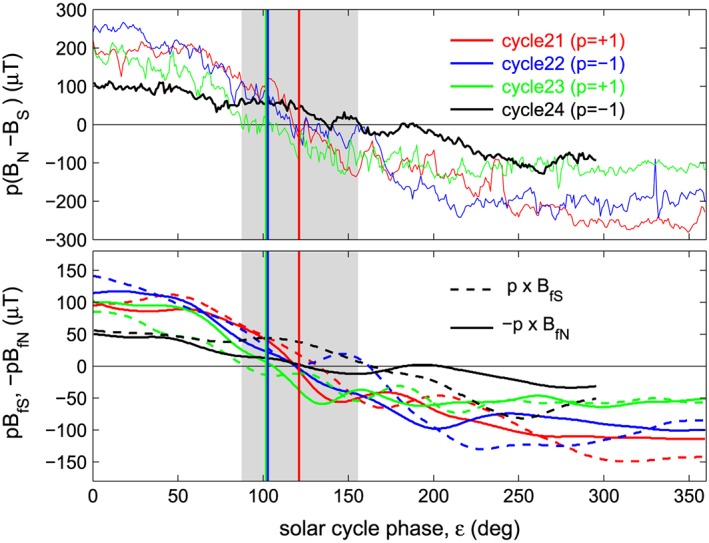
The solar polar fields observed by the magnetograph at Wilcox Solar Observatory (WSO) during solar cycles 21–24 (updated to the end of December 2016). (top) The difference between the two polar fields, *p(B*_N_ − *B*_S_ *)* as a function solar cycle phase, *ε*, as determined from the mean sunspot latitudes using the method described by *Owens et al.* [2011b], where *B*_N_ and *B*_S_ are the average fields seen over the north and south solar poles, respectively, and *p* = +1 for the odd‐numbered cycles and *p* = −1 for the even ones. The reversals in the polarity of the polar field difference all occur within the grey band and the phases of the peak sunspot number in 12 month running means are given by the vertical lines. (bottom) The −*pB*_fN_ (solid lines) and +*pB*_fS_ (dashed lines) as a function of *ε*, where *B*_fN_ and *B*_fS_ are the *B*_N_ and *B*_S_ data that have been passed through a 20 nHz low‐pass filter to remove the annual variation due to the heliographic latitude, Λ_H_, of Earth. In both panels, red, blue, green and black denotes solar cycles 21, 22, 23, and 24, respectively.

This behavior is compared to previous solar cycles in Figure 13. The timing of the polar field reversal, relative to sunspot maximum, was first observed during solar cycle 19 (SC19) by *Babcock* [1959] using data from the Hale Solar Laboratory (HSL) magnetograph. He noted that the average field emerging from the south solar pole reversed polarity between March and July 1957 and that in the north pole reversed in November 1958. The 12 month running mean of monthly sunspot number peaked in March 1958, midway between these two reversals. Figure 13 employs the continuous data on the solar polar field available from WSO [*Scherrer et al.,* 1977]. As noted by Babcock during SC19, the two poles do not reverse at exactly the same date, and the raw data are also complicated by a strong annual periodicity introduced by the annual variation in Earth's heliographic latitude. Because of these two effects, the average polar field reversals are most readily seen by taking the difference between the north and south fields, (*B*_N_ − *B*_S_): in this difference the annual variation in both *B*_N_ and *B*_S_, caused by the magnetograph's annual oscillation in Λ_H_, is canceled. In order to give the variations of this difference the same appearance in each sunspot number cycle (as opposed to Hale cycle), thereby allowing easy comparisons, Figure 13 (top) shows (*B*_N_ − *B*_S_) multiplied by *p*, where *p* = +1 for odd‐numbered cycles and *p* = −1 for even ones: the variation of *p*(*B*_N_ − *B*_S_) with solar cycle phase, *ε* (determined using the average of the absolute values of the northern and southern mean sunspot latitudes using the method of *Owens et al*. [2011a]) is plotted in the top panel for the WSO measurements, which are made every 10 days. The area shaded grey is between the earliest and latest reversals seen to date (lowest and largest *ε*). Figure 13 (bottom) shows −*pB*_fN_ and +*pB*_fS_ where *B*_fN_ and *B*_fS_ are the northern and southern polar field variations after they have been passed through a 20 nHz low‐pass filter to smooth them and remove the annual variation. The vertical lines give the phases of the corresponding cycle peaks in 12‐point running means of monthly sunspot numbers. Red, blue, and green are used to denote cycles 21, 22, and 23, and black is for 24.

At the time of writing, the magnitude of the polar field difference |*B*_N_ − *B*_S_| in cycle 24 is slightly lower than it was at the same phase of cycle 23. However, because of the decline in *B*_fS_ there has been a recent decline in this difference and this paper provides a reason to expect this decline to continue. No such decline was seen for cycle 23, and thus, this predicts that the polar field difference by the end of cycle 24 may well be slightly lower than it was at the end of cycle 23. This would make solar cycle 25 the slightly weaker than cycle 24, which would be consistent with the most likely prediction from the analogue forecasts based on cosmogenic isotope data made by *Barnard et al.* [2011]. At present (December 2016) |*B*_fN_| = 38 μT and is currently rising whereas |*B*_fS_| = 51 μT and is currently falling: if we assume they will become equal in magnitude by the end of cycle 24 (and Figure 13 shows that this has not always been the case for previous cycles), we would expect |*B*_fN_| ≈ |*B*_fS_| ≈ 45 μT. This can be compared with the values at the end of cycle 23 which were |*B*_fN_| = 51 μT and |*B*_fS_| ≈ 56 μT. The OSF panels in Figures 2, 3, 4 explain why this is likely to be the case, even though we have seen a large, and somewhat unexpected, increase in OSF during the declining phase of cycle 24: the reason is that much of the OSF emergence was hemispherically asymmetric and at times when the current sheet tilt was anomalously low. Both these conditions mean that the reconnection rate in HCS was low which causes temporary and large rise in OSF but does not advance the polar field polarity.
